# Gray matter atrophy rate as a marker of disease progression in AD

**DOI:** 10.1016/j.neurobiolaging.2010.11.001

**Published:** 2012-07

**Authors:** Valerie M. Anderson, Jonathan M. Schott, Jonathan W. Bartlett, Kelvin K. Leung, David H. Miller, Nick C. Fox

**Affiliations:** a Department of Neuroinflammation, UCL Institute of Neurology, Queen Square, London, UK; b Dementia Research Centre, UCL Institute of Neurology, Queen Square, London, UK; c Department of Medical Statistics, London School of Hygiene and Tropical Medicine, London, UK

**Keywords:** Gray matter, Atrophy rate, Alzheimer's disease, Sample sizes, MRI

## Abstract

Global gray matter (GM) atrophy rates were quantified from magnetic resonance imaging (MRI) over 6- and 12-month intervals in 37 patients with Alzheimer's disease (AD) and 19 controls using: (1) nonlinear registration and integration of Jacobian values, and (2) segmentation and subtraction of serial GM volumes. Sample sizes required to power treatment trials using global GM atrophy rate as an outcome measure were estimated and compared between the 2 techniques, and to global brain atrophy measures quantified using the boundary shift integral (brain boundary shift integral; BBSI) and structural image evaluation, using normalization, of atrophy (SIENA). Increased GM atrophy rates (approximately 2% per year) were observed in patients compared with controls. Although mean atrophy rates provided by Jacobian integration were smaller than those from segmentation and subtraction of GM volumes, measurement variance was reduced. The number of patients required per treatment arm to detect a 20% reduction in GM atrophy rate over a 12-month follow-up (90% power) was 202 (95% confidence interval [CI], 118–423) using Jacobian integration and 2047 (95% CI 271 to > 10 000) using segmentation and subtraction. Comparable sample sizes for whole brain atrophy were 240 (95% CI, 142–469) using the BBSI and 196 (95% CI, 110–425) using SIENA. Jacobian integration could be useful for measuring GM atrophy rate in Alzheimer's disease as a marker of disease progression and treatment efficacy.

## 1 Introduction

Alzheimer's disease (AD) is a neurodegenerative disease characterized by progressive cognitive decline. With potential disease-modifying therapies for AD being developed, sensitive, objective, and reliable markers of disease progression and therapeutic effects are crucial. Measurements of whole brain and hippocampal atrophy rates from serial structural magnetic resonance imaging (MRI) are potential markers of the underlying neuroaxonal damage and disease progression in AD (Fox et al., 2005; Henneman et al., 2009; Morra et al., 2009; Sluimer, et al., 2008; Whitwell, et al., 2008).

Gray matter (GM) may lose volume earlier in AD than white matter (WM) (Serra, et al., 2010; Tanabe et al., 1997), and GM loss has been shown to be associated with ongoing pathological and clinical progression of the disease (Whitwell et al., 2008; de Jong et al., 2008; Mouton et al., 1998; Serra et al., 2010), and may therefore be a more sensitive marker of AD pathology than whole brain atrophy.

Despite these observations few studies have specifically investigated rates of global GM atrophy in AD, which may be a useful marker of disease progression in clinical trials. Optimizing the power of outcome measures for clinical trials is important, as the number of subjects required to show a therapeutic effect on progression may be reduced, thereby leading to more efficient trials and exposing fewer patients to possible side effects. A common method for quantifying GM atrophy is segmentation and subtraction of serial GM volumes (Calabrese et al., 2009; Cardenas et al., 2003; Moore et al., 2009). However nonlinear registration of magnetic resonance (MR) images, which determines a deformation field to match serial scans, could be used to quantify directly an individual's gray matter (GM) structural changes over time (Freeborough and Fox, 1998; Hua et al., 2009), and provide a measure that is more precise and which would therefore have greater power to detect change — largely because the influence of segmentation errors at each time point is reduced (Anderson et al., 2007a, 2007b). The aim of this study was to determine global GM atrophy rates in patients with AD and controls over periods of 6- and 12-months, using: (1) nonlinear registration and Jacobian integration, and (2) segmentation and subtraction of serial GM volumes, and to estimate the statistical power of these 2 techniques in potential clinical trials using global GM atrophy rate as an outcome measure. For comparison, whole brain atrophy rates were also calculated using 2 registration-based methods (the brain boundary shift integral; BBSI) (Fox and Freeborough, 1997; Freeborough and Fox, 1997) and structural image evaluation, using normalization, of atrophy (SIENA) (Smith et al., 2002).

## 2 Methods

Thirty-seven patients with probable AD, recruited from the Cognitive Disorders Clinic at the National Hospital for Neurology and Neurosurgery, and 19 control subjects were included in the study. This cohort has been the subject of a previous report where full details of inclusion criteria and clinical assessment have been given (Schott et al., 2005). One individual recruited to the original study has since had a postmortem diagnosis of Lewy body dementia, and was therefore excluded from the current study. Three other patients have had postmortem confirmation of a diagnosis of AD. The study was granted ethical approval by the National Hospital for Neurology and Neurosurgery and Institute of Neurology joint Research Ethics Committee, and subjects gave written informed consent.

### 2.1 MRI scan acquisition

All subjects underwent MRI scanning on a 1.5 T Signa scanner (GE Medical Systems, Milwaukee, WI, USA) at baseline and at approximately 6 months and 12 months (mean intervals 180 days, SD 7; and 365 days, SD 14). T1-weighted volumetric images were obtained using an inversion recovery prepared fast spoiled gradient echo sequence with acquisition parameters time to repetition = 15 ms, time to echo = 5.4 ms, flip angle = 15°, TI = 650 ms, a 24-cm field of view and a 256 × 256 matrix, to provide 124 contiguous 1.5-mm thick slices in the coronal plane (voxels 0.9735 mm × 0.9735 mm × 1.5 mm).

### 2.2 MRI scan processing

Images were corrected for intensity inhomogeneity using the N3 algorithm (www.bic.mni.mcgill.ca/software/N3/) (Sled et al., 1998), and the images were segmented into brain/nonbrain using a semiautomated technique (MIDAS) (Freeborough et al., 1997). Two methods for quantifying rates of global gray matter atrophy and 2 methods for quantifying rates of whole brain atrophy were subsequently applied over 6- and 12-month intervals as follows.

#### 2.2.1 Segmentation and subtraction of serial GM volumes

**Fig. 1:**
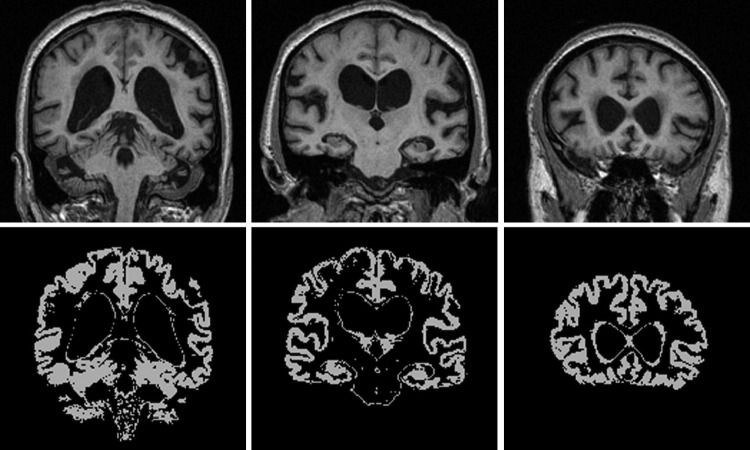
Example binary gray matter segmentation provided by SPM5 (http://www.fil.ion.ucl.ac.uk/spm/; Wellcome Trust Centre for Neuroimaging, UCL Institute of Neurology, London, UK), which includes subcortical gray matter structures.

All images and brain regions were transformed into Montreal Neurological Institute 305 atlas space following a 12-degrees of freedom registration, but applying only 6 dof (translations and rotations), and images were resampled to produce isotropic voxels (1 × 1 × 1 mm^3^). Baseline and repeat images in Montreal Neurological Institute 305 space were segmented into GM, WM, and cerebrospinal fluid using SPM5 (http://www.fil.ion.ucl.ac.uk/spm/; Wellcome Trust Centre for Neuroimaging, UCL Institute of Neurology, London, UK) (Ashburner and Friston, 2005). The resulting global GM probability maps were transformed into binary masks (Fig. 1) by applying a threshold which included voxels that had a probability ≥ 0.5 into the final image. The volumes of the binary GM masks were determined and global GM atrophy was quantified as (baseline GM volume) - (repeat GM volume).

#### 2.2.2 Nonlinear registration and Jacobian integration over GM

The 6- and 12-month repeat images in standard atlas space were registered to baseline using a 12-dof affine registration (Woods et al., 1998), and the intensity of the baseline and repeat images were normalized to each other. The baseline brain region, morphologically dilated 3 times, was used to crop baseline and registered repeat images. Using the result of the affine registration, a nonlinear fluid registration was applied to warp the cropped repeat image to the cropped baseline image (Freeborough and Fox, 1998). The fluid algorithm iteratively drove the deformation field to maximize the cost function (cross correlation) of the voxels while forcing the deformation field to satisfy the compressible viscous fluid model. At each iteration, the body force for the fluid equation was calculated as the derivative of the cost function. Exit criteria were satisfied when the mean body force fell below a threshold of 5.0 × 10^−8^ (a value based on optimization of the determinant of the Jacobian matrix in a subset of 6 controls and 6 patients in whom we ran the nonlinear registration for 1500 iterations), and windowed sinc interpolation was used to generate the final registered repeat image. All fluid registrations were visually inspected to ensure there was adequate matching of the baseline and fluidly-registered repeat images. The resulting deformation field allowed the Jacobian matrix for each voxel to be obtained. The determinant of these matrices describes the expansion (>1) and contraction (<1) at each voxel. GM regions of interest were generated from the SPM5 binary baseline GM images, and global GM atrophy was quantified by integrating the Jacobian values of expansion and contraction within these regions.

#### 2.2.3 Brain boundary shift integral

The original 6- and 12-month repeat images in standard atlas space were registered to baseline using a 12-dof affine registration (Woods et al., 1998). The repeat brain region and image were resliced using the transformation parameters obtained. The intensity of the baseline and repeat images were normalized to each other by performing a linear regression of the intensity of cerebrospinal fluid, GM, WM, and the brain between the baseline and repeat images (Leung et al., 2010). From each registered image pair the brain volume change was calculated by integrating the sampled difference of brain voxel intensities over a region defined as the intersection of the baseline and repeat brain regions dilated by 1 voxel minus the intersection of the baseline and repeat region eroded by 1 voxel (Freeborough and Fox, 1997). The intensity window was automatically determined by the intensity of CSF and GM in the baseline and repeat images (Leung et al., 2010).

#### 2.2.4 SIENA

Percentage brain volume change between the 6- and 12-month time points and baseline was estimated with SIENA (Smith et al., 2001, 2002). The brain regions obtained from the initial segmentation of images using MIDAS were utilized, but SIENA was used extract a skull image from all scans (Smith, 2002). Baseline and repeat brain images were then aligned to each other using the skull images to constrain the registration scaling (Jenkinson and Smith, 2001; Jenkinson et al., 2002), and both baseline and repeat brain images were resampled into the space halfway between the 2. Tissue-type segmentation was carried out in order to find brain/nonbrain edge points (Zhang et al., 2001), and then perpendicular edge displacement between the 2 time points was estimated at these edge points. Finally, the mean edge displacement was converted into a global estimate of percentage brain volume change between the 2 time points.

#### 2.3 Statistical analysis

Statistical analyses were performed using STATA 11 (StataCorp, College Station, TX, USA). Atrophy was expressed as a percentage of baseline volume and annualized. Atrophy rates are expressed as a positive number (and “growth” expressed as a negative number). Comparisons within subject groups between GM atrophy rates estimated using the 2 techniques were made by calculating the mean of the paired differences, and by calculating a 95% confidence interval (CI) assuming normality of these differences. Differences in the variances of GM atrophy rates were compared by reporting the ratio of the SDs, with 95% CIs found using Pitman's method. Linear regression analyses were used to compare GM atrophy rates between the control and patient groups using a group indicator and adjusting for age and gender as covariates.

Sample size calculations were performed for a trial including baseline and 1 follow-up MRI, and were based on the standard formula (Fox et al., 2000; Kirkwood, 1988), with 90% power to detect a 20% reduction in either global GM or whole brain atrophy rate at the 5% 2-tailed significance level.

$$\text{Sample}\;\text{size}\;\text{per}\;\text{trial}\;\text{arm=}{\left(\text{u+v}\right)}^{\text{2}}\left({\text{2σ}}^{\text{2}}\right)\text{/}{\left({\text{μ}}_{\text{1}}{\text{-μ}}_{\text{2}}\right)}^{\text{2}}$$

u = 1.28 to provide 90% power; v = 1.96 to test at the 5% significance level. μ_1_ and μ_2_ are the mean GM or brain atrophy rates in the placebo and treatment groups. σ^2^ is the variance of the GM or brain atrophy rate (the variance in the patient group).

Calculations were performed both without taking normal aging into account (assuming a completely effective treatment would reduce the atrophy rate to 0), and allowing for normal aging (assuming a completely effective treatment would reduce the atrophy rate to the rate observed in controls). The mean rate in the treatment group was taken as a percentage of the difference between the control atrophy rate (or 0 when not taking aging into account) and the atrophy rate in patients with AD. An immediate and constant treatment effect was assumed. The effect of the atrophy measurement technique on sample sizes was assessed by determining the ratios of sample sizes, with 95% bias-corrected and accelerated bootstrap CIs calculated (10,000 bootstrap samples) to indicate the precision with which the ratios had been estimated.

## 3 Results

The AD and control subjects were well matched for age (AD mean 69.6 years [SD 7.2], control mean 69.3 years [SD 7.1]), while there was a smaller proportion of males in the AD group compared with controls (ad 14 males:23 females; controls 10 males:9 females).

**Fig. 2:**
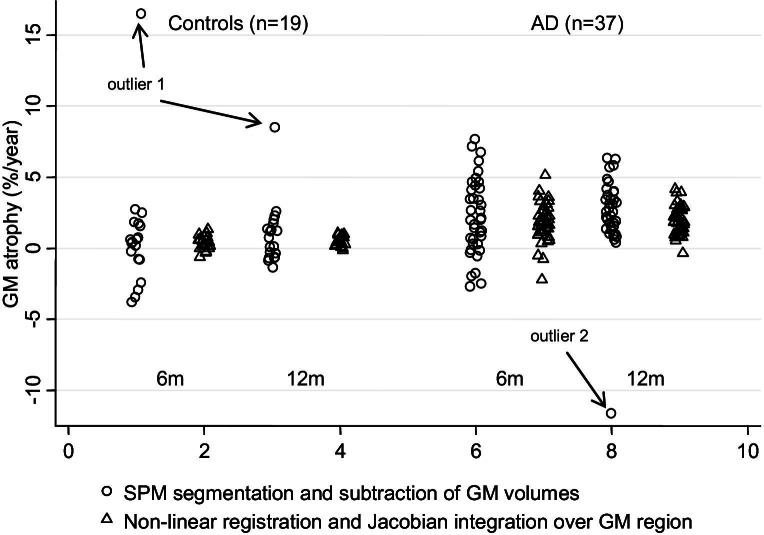
Gray matter (GM) atrophy rates in Alzheimer's disease (AD) and control subjects. Gray matter atrophy rates (% per year) in patients with Alzheimer's disease and control subjects calculated over intervals of 6 and 12 months using: (1) SPM5 segmentation and subtraction of GM volumes, and (2) nonlinear registration and Jacobian integration over GM regions. Arrows indicate the measurements from 2 subjects (1 control, 1 ad) for whom the segmentation and subtraction estimates of atrophy are markedly outside of the expected range.

Annualized GM and whole brain atrophy rates for patients with AD and control subjects using the different techniques are reported in Table 1. Fig. 2 shows the annualized GM atrophy rates using the 2 different techniques and suggests that SPM5 segmentation and subtraction produced larger than expected GM volume changes in 1 control subject (rate of GM atrophy 16.5% per year using the 6-month scan and 8.7% per year using the 12-month scan), and 1 patient with AD (rate of GM atrophy −11.6% per year using the 12-month scan). The images and GM segmentations of these subjects showed no apparent problems on visual inspection. To examine their influence on the results for segmentation and subtraction, we present results of the analyses both including and excluding them.

Mean annualized GM atrophy rates were similar at both 6 and 12 months. In controls and patients, Jacobian integration gave lower mean atrophy rates than segmentation and subtraction (when including the 2 outliers) over both 6-month and 12-month intervals, although none of the differences were statistically significant. The SD of the annual rates found by Jacobian integration in controls was 0.12 (95% CI, 0.08–0.20, *p* < 0.001) that of the SD of the segmentation and subtraction rates. In the AD group the corresponding ratio of SDs was 0.34 (95% CI, 0.25–0.46, *p* < 0.001).

Mean baseline GM volume was 653 mL (SD 53) in controls, and 540 mL (SD 69) in patients with AD (mean difference adjusted for age and gender was 108 mL, 95% CI, 74–142, *p* < 0.001). The mean differences in GM atrophy rates between patients and controls were similar when calculated over 6- or 12-month intervals using each technique; mean patient-control difference (adjusted for age and gender) measured over the 12-month interscan interval was 1.44% per year (95% CI, -0.10 to 2.98, *p* = 0.067) using segmentation and subtraction and 1.54% per year (95% CI, 1.08–2.00, *p* < 0.001) using Jacobian integration. A greater difference in GM atrophy rates between patients and controls was observed with segmentation and subtraction when excluding the 2 subjects with large volume changes (mean difference over the 12-month interval was 2.21% per year, 95% CI, 1.32–3.11, *p* < 0.001).

Table 1 also shows the estimated sample size requirements for trials with 90% statistical power to detect a 20% reduction in GM or whole brain atrophy rate, either with or without allowing for aging. The estimated sample sizes were smaller when using Jacobian integration to quantify GM atrophy compared with segmentation and subtraction: for a 6-month trial the estimated sample size using Jacobian integration (allowing for aging) was 0.26 (95% CI, 0.01–1.20) that of the sample size using segmentation and subtraction, although this was not statistically significant. For a 12-month trial the corresponding ratio was 0.10 (95% CI, 0.01–0.72) which was statistically significant. The sample size estimates for segmentation and subtraction for a 12-month trial were influenced to a large extent by the 2 subjects with large volume changes quantified using segmentation and subtraction — excluding these subjects reduced the sample size estimates to levels similar (but still larger) than those for Jacobian integration. Estimated sample sizes were similar for global GM atrophy rate measured using Jacobian integration to whole brain atrophy rate measured by either the BBSI or SIENA.

## 4 Discussion

In this study we assessed global GM atrophy rates from serial MRI over 6- and 12-month intervals in probable AD and control subjects, using 2 different measurement techniques. First we used the standard approach of measuring GM volumes on each scan separately (using SPM5) and then calculated the atrophy by subtracting the second value from the first. Second, we used a “direct” measure derived from nonlinear registration of each pair of scans and then integration of the Jacobian values over the global GM region. We compared these measurements both to each other and to 2 whole brain atrophy measures that are currently utilized in clinical trials in AD, namely the BBSI and SIENA. We found evidence that GM atrophy is greater in patients with AD compared with controls (∼4 times greater) over intervals as short as 6 months. Furthermore, we have demonstrated that Jacobian integration reduces variability and may offer increased statistical power compared with segmentation and subtraction of serial GM volumes, and that it offers similar statistical power to whole brain atrophy rates measured by the BBSI and SIENA. In addition, Jacobian integration may be a more robust technique for measuring GM atrophy than SPM5 segmentation and subtraction which produced what appeared to be unexplained and erroneous results in 2 subjects.

Widespread involvement of the GM (cortical and deep GM) in AD has been shown using statistical mapping techniques and specific region-of-interest analyses on MRI (de Jong et al., 2008; Serra et al., 2009; Singh et al., 2006). However, it has been demonstrated that atrophy rates within the cortex are not uniform either spatially or temporally in patients with mild cognitive impairment (MCI) and AD (McDonald et al., 2009; Thompson et al., 2003; Whitwell et al., 2007). It has been suggested that analyzing larger regions of the brain may be more powerful (because of precision issues) than using small regions (Hua et al., 2008a), and our study investigated global GM atrophy as a marker of disease progression, which may be influenced less by disease stage or severity than more localized cortical region-of-interest measures.

Using 2 different techniques for measuring global GM atrophy, we demonstrated a substantial loss of GM in patients with AD over an interval as short as 6 months, and the annual atrophy rate provided by segmentation and subtraction was similar to that derived using similar methodology for the cortex in a previous study of patients with dementia (2.4% per year [SD 2.9] vs. 2.8% per year [SD 1.8]) (Cardenas et al., 2003). The mean GM atrophy rate provided by Jacobian integration was lower than that provided by segmentation and subtraction in our study. The Jacobian integration technique used in this study has been applied previously to determine the rate of hippocampal atrophy in patients with AD. Similarly to our study, lower atrophy rates were obtained using Jacobian integration compared with segmentation and subtraction of serial hippocampal volumes (Barnes et al., 2007, 2008). Underestimation of global brain atrophy was also found when applying this Jacobian integration technique to MRI on which atrophy has been simulated (Camara et al., 2008). One reason that may underlie this finding could be the inclusion of partial volume and CSF voxels in the region of interest, in our case the baseline GM. These voxels may increase in volume over time as the brain atrophies, partially negating any real GM loss that has occurred. In addition, small inaccuracies in the registration of images or interpolation could account for errors in the calculation of the deformation field.

Nonlinear registration and Jacobian integration has been utilized previously in patients with MCI and AD in both cross-sectional and longitudinal studies (also called tensor-based morphometry) (Hua et al., 2008a, 2008b; Leow et al., 2009; Studholme et al., 2004). These studies have used the calculated deformations to examine the distribution of atrophy at a group level, and when atrophy has been quantified, it has been limited to smaller regions-of-interest defined on template images, rather than global scan-specific segmentations. Moreover, these studies have not investigated the ability of these techniques to measure atrophy relative to other manual or automated measures. However, 1 study investigating tissue growth in the WM and GM of infants reported a general agreement between estimates provided by a nonlinear registration and Jacobian integration technique and those provided by segmentation and subtraction of volumes (Aljabar et al., 2008). The discrepancy with the results of the comparison in our study may be explained by the fact that growth rather than atrophy was being quantified, and also that different nonlinear registration and segmentation algorithms were being used (free-form deformation and expectation maximization respectively).

The mean rates of atrophy provided by Jacobian integration were lower than those from segmentation and subtraction (although not statistically significantly), but there was evidence that variance was reduced using this technique, presumably due to reduced measurement error. It must also be noted that segmentation and subtraction produced 1 or 2 extreme outliers (Fig. 2), which were neither plausible nor consistent over time. While it may have been possible to optimize the images or segmentation process in these 2 subjects to rectify any problems which may have led to these results, 1 of the advantages of the Jacobian (registration-based) method seems to be a reduction in the potential for large errors in measurement of change. This may be advantageous in clinical trials where minimizing the need for intervention in the case of erroneous results is beneficial when processing hundreds of images. Power calculations estimating patient numbers for therapeutic trials depend both on the difference in means between treatment arms and the variance of measured atrophy, and consequently, the sample size estimates for a placebo-controlled trial using GM atrophy rate as an outcome measure were consistently smaller using the Jacobian integration technique than segmentation and subtraction. Interestingly, GM Jacobian integration provided relatively similar statistical power to whole brain atrophy measures from the BBSI and SIENA (as previously reported in this patient population, very similar results were seen using either the BBSI or SIENA; Smith et al., 2007). However GM atrophy may be more disease-specific and clinically relevant than whole brain atrophy or ventricular enlargement, given the density of neuronal cell bodies in the GM and the associations that have been shown between GM atrophy and cognition (de Jong et al., 2008; Mouton et al., 1998). It may well be that the GM-focused measures might have particular advantages in early disease. For example GM atrophy, but not WM atrophy, has been observed in people with amnestic MCI (Balthazar et al., 2009), while evidence of a sequential relationship between hippocampal atrophy and WM pathology in early AD has been reported (Villain et al., 2010). However, the postulated gain in using our proposed GM atrophy rate measurement method compared with methods measuring whole brain atrophy rates in MCI and early AD requires further investigation.

It should be noted that in addition to sample size estimates taking normal aging into account, we provided estimates without taking normal aging into account, to enable comparisons with previously published data using similar nonlinear registration and Jacobian integration techniques. A study based on atrophy rates in the temporal lobe provided by tensor-based morphometry reported sample size estimates ranging from 70 to 104 subjects per treatment arm, depending on the parameters used for nonlinear registration, for a 25% reduction in atrophy rate and 90% power (Hua et al., 2009). It is possible to extrapolate the sample sizes estimated in our study to other effect sizes by multiplying by the square of the ratio of the effect sizes. Therefore, using the Jacobian integration methodology, an estimated 77 patients per treatment arm would be required to detect a 25% reduction in GM atrophy rate in our study (120 subjects multiplied by 16/25; i.e., 4/5 squared). These sample sizes compare well with those derived using similar methods over the temporal lobe in Hua et al. (2009). However, it is important to take normal aging into account, or the potential for a therapeutic effect may be overestimated (Schott et al., 2010); in this instance, 130 subjects per treatment arm would be required for a 25% effect size and 90% power when taking normal aging into account.

Although this study has shown that global GM atrophy could be used to track disease progression in patients with AD, 1 limitation of the study was that we did not look at the association of GM atrophy with clinical outcomes, which could have strengthened the evidence for our hypothesis that GM atrophy may be more disease-specific and clinically relevant than measures of whole brain atrophy or ventricular enlargement in patients with AD. Furthermore, this was a preliminary study, and additional validation and optimization of the nonlinear registration and Jacobian integration technique should be performed. Although we used a 0.5 probability of being GM for classification of the baseline GM mask, other probability thresholds should be investigated, as misclassified or partial volume voxels will increase measurement error. This study used SPM5 to classify the different tissues, but SPM8 is now available which includes new segmentation methodology that may offer a more accurate segmentation of the convoluted cortex. This may have offered improved results in the 2 subjects who gave results which were inconsistent with what we expected. Additionally, it should be investigated whether erosion of the resulting GM masks could improve the sensitivity and precision of atrophy measurement. As mentioned previously, other nonlinear registration algorithms are available and could also be investigated (Christensen et al., 1996; Rueckert et al., 1999; Shen and Davatzikos, 2003), as it has been shown that nonlinear registration algorithms can differ in their accuracy and reproducibility (Klein et al., 2009; Yanovsky et al., 2009). Similarly, techniques which attempt to compute temporally consistent segmentations for longitudinal atrophy assessment by jointly segmenting serial volumes have been proposed, and the relative merits of these methods should be compared with that presented in the current study (Xue et al., 2006; Wolz et al., 2010). It has also been shown that the reliability of nonlinear registration and quantification of deformations can be affected by the pulse sequence, coil-type and postprocessing, and these factors, and the parameters of the nonlinear registration, should be optimized in any future prospective studies utilizing these techniques (Leow et al., 2006). Additionally, future work should investigate the application to patients with MCI and neurological disorders other than AD, such as multiple sclerosis, in which there is currently considerable interest in GM pathology.

We conclude that nonlinear registration and integration of Jacobian values has the potential to track GM atrophy in patients with AD from serial MRI, and provide similar statistical power to currently used registration-based whole-brain atrophy measures, and increased statistical power compared with segmentation and subtraction of serial GM volumes. These results may have implications for future clinical trials of disease-modifying treatments in AD.

## Disclosure statement

Dr. Fox has served on the scientific advisory boards of Alzheimer's Research Forum, Alzheimer's Society, and Alzheimer's Research Trust, and editorial boards of *Alzheimer's Disease and Associated Disorders; Neurodegenerative Diseases,* and *BioMed, Central — Alzheimer's Research and Therapy*. He holds a patent for QA Box that may accrue revenue. In the last 5 years his research group has received payment for consultancy or for conducting studies from Abbott Laboratories, Elan Pharmaceuticals, Eisai, Eli Lilly, GE Healthcare, IXICO, Lundbeck, Pfizer, Inc., Sanofi-Aventis, and Wyeth Pharmaceuticals. He receives research support from MRC (G0801306 [PI], G0601846 [PI]), NIH (U01 AG024904 [Coinvestigator] [sub contract]), Alzheimer Research Trust (ART/RF/2007/1 [PI]), NIHR (Senior Investigator) and EPSRC (GR/S48844/01 [PI]). The other authors report no conflicts of interest.

The study was granted ethical approval by the National Hospital for Neurology and Neurosurgery and Institute of Neurology joint Research Ethics Committee, and subjects gave written informed consent.

## Figures and Tables

**Table 1:** Mean (SD) annualized gray matter and brain atrophy rates

	Mean (SD) atrophy (% per year)		Sample size (95% CI)	
	Controls (*n* = 19)	AD (*n* = 37)	Not allowing for aging	Allowing for aging
6 months				
Gray matter atrophy				
Segmentation and subtraction				
All subjects	0.86 (4.24)	2.20 (2.66)	771 (376–2322)	2081 (413 to > 10000)
Excluding outliers (1 control, 1 AD)	−0.01 (1.97)	2.31 (2.60)	666 (336–1941)	661 (255–3284)
Jacobian integration	0.42 (0.47)	1.77 (1.37)	314 (162–953)	540 (243–2252)
Whole brain atrophy				
BBSI	0.51 (0.57)	1.67 (1.26)	296 (156–668)	617 (257–2184)
SIENA	0.34 (0.89)	2.03 (1.63)	337 (178–871)	485 (216–1685)
1 year				
Gray matter atrophy				
Segmentation and subtraction				
All subjects	0.92 (2.21)	2.37 (2.86)	763 (158 to > 10,000)	2047 (262 to > 10,000)
Excluding outliers (1 control, 1 AD)	0.49 (1.19)	2.76 (1.64)	184 (125–282)	273 (151–617)
Jacobian integration	0.46 (0.27)	2.01 (0.96)	120 (75–221)	202 (119–420)
Whole brain atrophy				
BBSI	0.64 (0.44)	1.99 (0.91)	110 (75–167)	240 (142–469)
SIENA	0.67 (0.82)	2.72 (1.25)	111 (70–178)	196 (110–425)

Mean (SD) global gray matter atrophy rates (% per year) in patients with AD and control subjects calculated over intervals of 6 and 12 months using: (1) SPM5 segmentation and subtraction of GM volumes, and (2) nonlinear registration and Jacobian integration over GM regions. Also included are the global brain atrophy rates quantified using the BBSI and SIENA. Estimated sample sizes (95% CIs) for each method are also given for a placebo-controlled clinical trial to provide 90% power to detect a 20% reduction in atrophy at the 5% significance level, either with or without allowing for aging. Alternative effect sizes can be extrapolated from the results given by multiplying by the square of the ratio of the effect sizes, e.g., for a 25% effect size, the estimates given in the table would be multiplied by 4/5 squared, or 16/25. For sample sizes based on 80% power, estimates should be multiplied by 0.747. Key: AD, Alzheimer's disease; BBSI, brain boundary shift integral; CI, confidence interval; GM, gray matter; SIENA, structural image evaluation, using normalization, of atrophy.
